# AC133+ progenitor cells as gene delivery vehicle and cellular probe in subcutaneous tumor models: a preliminary study

**DOI:** 10.1186/1472-6750-9-28

**Published:** 2009-03-27

**Authors:** Ali M Rad, ASM Iskander, Branislava Janic, Robert A Knight, Ali S Arbab, Hamid Soltanian-Zadeh

**Affiliations:** 1 Department of Radiology, Henry Ford Hospital, Detroit, Michigan, USA; 2 Department of Radiology, Massachusetts General Hospital/Harvard Medical School, Boston, MA, USA; 3 Department of Neurology, Henry Ford Hospital, Detroit, Michigan, USA; 4 Control and Intelligent Processing Center of Excellence, Department of Electrical and Computer Engineering, University of Tehran, Tehran, Iran

## Abstract

**Background:**

Despite enormous progress in gene therapy for breast cancer, an optimal systemic vehicle for delivering gene products to the target tissue is still lacking. The purpose of this study was to determine whether AC133+ progenitor cells (APC) can be used as both gene delivery vehicles and cellular probes for magnetic resonance imaging (MRI). In this study, we used superparamagentic iron oxide (SPIO)-labeled APCs to carry the human sodium iodide symporter (hNIS) gene to the sites of implanted breast cancer in mouse model. In vivo real time tracking of these cells was performed by MRI and expression of hNIS was determined by Tc-99m pertechnetate (Tc-99m) scan.

**Results:**

Three million human breast cancer (MDA-MB-231) cells were subcutaneously implanted in the right flank of nude mice. APCs, isolated from fresh human cord blood, were genetically transformed to carry the hNIS gene using adenoviral vectors and magnetically labeled with ferumoxides-protamine sulfate (FePro) complexes. Magnetically labeled genetically transformed cells were administered intravenously in tumor bearing mice when tumors reached 0.5 cm in the largest dimension. MRI and single photon emission computed tomography (SPECT) images were acquired 3 and 7 days after cell injection, with a 7 Tesla animal MRI system and a custom built micro-SPECT using Tc-99m, respectively. Expression of hNIS in accumulated cells was determined by staining with anti-hNIS antibody. APCs were efficiently labeled with ferumoxide-protamine sulfate (FePro) complexes and transduced with hNIS gene. Our study showed not only the accumulation of intravenously administered genetically transformed, magnetically labeled APCs in the implanted breast cancer, but also the expression of hNIS gene at the tumor site. Tc-99m activity ratio (tumor/non-tumor) was significantly different between animals that received non-transduced and transduced cells (P < 0.001).

**Conclusion:**

This study indicates that genetically transformed, magnetically labeled APCs can be used both as delivery vehicles and cellular probes for detecting *in vivo *migration and homing of cells. Furthermore, they can potentially be used as a gene carrier system for the treatment of tumor or other diseases.

## Background

Breast cancer is the leading cause of death among women [1,2]. Against substantial research over the past decades, no definitive method for early detection and treatment of the breast cancer has been found. Gene therapy holds enormous therapeutic potential for breast cancer treatment [3-12]. In cancer gene therapy, viral vectors are administered locally or systematically [13]. Gene therapy using replication competent viral vectors could deliver their effects in two ways: 1) viral vectors can kill the tumor cells by infecting them and replicating inside cells (oncolysis); or 2) viral vectors can be used to insert therapeutic or suicidal genes, which can be targeted later [11,14]. When administered locally, however, the infiltrative nature of the breast cancer poses a problem for the successful delivery of genes to the sites of invading tumor cells. In addition, several factors, including lack of an efficient vector and delivery system, limit the effectiveness of systemically delivered genes. Very recently, progenitor cells have been used as carriers for therapeutic genes and are considered as delivery vehicles for transferring exogenous genes to the cancer cells [15,16]. In both cases, it is necessary to monitor the migration and homing of the genetically modified cells. However, current *in vivo *imaging techniques lack the ability to track the real time migration and homing of genetically altered cells to cancer tissue with acceptable resolution.

Labeling of cells with ferumoxides or other superparamagnetic iron oxides (SPIO) to track the migration of labeled cells is becoming routine in cellular MRI [17-22]. Recently, we have been able to magnetically label different mammalian cells including hematopoietic stem cells using two Food and Drug Administration (FDA) approved agents: ferumoxide and protamine sulfate. Ferumoxide is an incomplete dextran coated SPIO agent with an extremely high T2-relaxivity. It is used as contrast agent for liver diseases [23,24]. We have shown a linear correlation between relaxivity and iron concentration in labeled cells [25]. Protamine sulfate is used clinically as antidode for heparin toxicity. It has been shown that viability, functionality, and differentiation capacity of labeled cells (including stem cells) do not change after labeling [19,26].

AC133+ cells are a subpopulation of CD34+ hematopoietic stem cells, which are believed to be more pluripotent, and incorporate into endothelial lining of neovessels in normal and pathological conditions [27]. The purpose of this study was to determine whether AC133+ progenitor cells (APCs) can be used as gene delivery/carrier vehicles and as cellular probes for MRI. We used superparamagentic iron oxide (SPIO)-labeled AC133+ progenitor cells (APCs) to carry the hNIS gene to sites of implanted breast cancer in a mouse model. *In vivo *real time tracking of cells was performed by MRI and expression of hNIS was determined by Tc-99m pertechnetate (Tc-99m) scan.

## Results

Our experiments and data from other published papers indicate that MDA-MB-231 is a slow growing breast cancer cell line. Tumor Xenografts will appear in nude mice 6 to 7 days after inoculation and grow to 0.5 to 0.7 cm in 14 days [28]. Our data showed that the tumor can have internal bleeding or surface ulceration when it grows to more than 1.2 cm in a short period of time. Our main focus was to track the administered APCs at the sites of active angiogenesis in xenoplanted breast cancer, which is not related to the presence or absence of estrogen receptor.

### Efficiency of Iron Labeling

Labeling efficiency with FePro complexes was more than 90%, as determined by manual counting of PB-stained and unstained cells using a microscope.

### Mean Iron Concentration

Mean intra-cellular iron concentration was measured based on a spectrophotometric method as described in the Method Section. The iron concentration was determined from three different batches of labeled cells, which were selected randomly during our experiments. The iron concentration was 8.4 ± 2.8 pg per cell.

### Viability of Labeled Cells

Magnetically labeled genetically transduced APCs showed more than 75% viability compared to unlabeled non-transduced cells, whereas control unlabeled or labeled APCs were more than 90% viable.

### Efficiency of Viral Transduction

**Figure 1:**
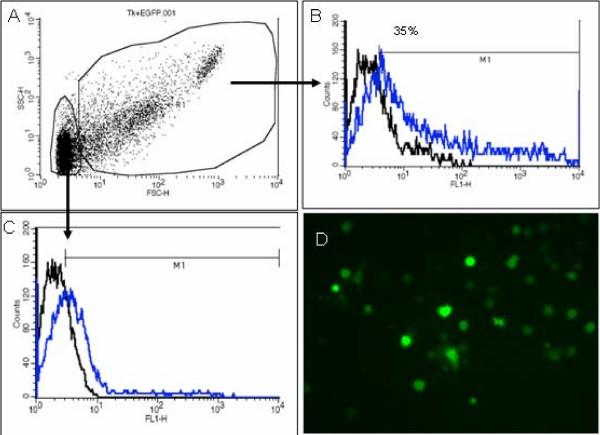
**Transfection efficiency using adenoviral vectors containing EGFP as a reporter gene**. Flowcytometric analysis shows 30% to 35% EGFP positive cells in both cell populations. Fluorescent microscopy also shows EGFP positive cells in culture even after 12 days of infection.

Flowcytometric analysis indicated around 35% transduction efficiency using adenoviral vectors containing EGFP gene (Figure 1). Follow up fluorescent microscopic examination indicated substantial number of EGFP positive cells even after 12 days (Figure 1).

### MRI Results

**Figure 2:**
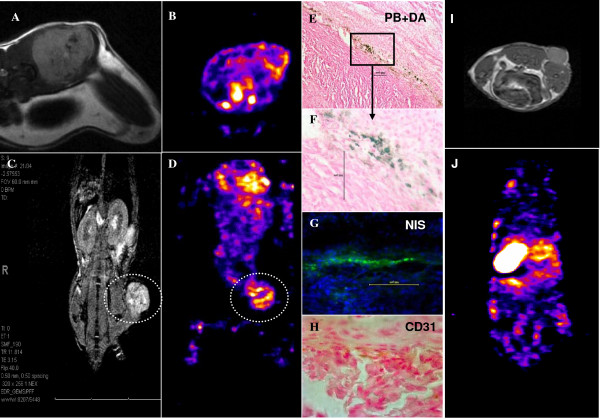
**Accumulation of magnetically labeled, transgenic AC133+ progenitor cells (APCs) around the implanted tumor**. MRI shows low signal intensity areas at the margin of the tumor (A, C), which are at the corresponding sites of iron positive cells detected by Prussian blue staining (E, F). The central low signal intensity areas are due to hemorrhagic foci within the tumor. B (trans-axial sections) and D (coronal sections) of SPECT studies indicate the accumulated transgenic APCs that are detected by T-99m (within the white dotted oval ROI). The SPECT study also proves the migration and homing of APCs at the margin of the tumors (seen on MRI). Immunohistochemistry shows the accumulation of NIS positive cells and CD31 positive cells at the corresponding sites, as detected by labeled secondary antibodies (G, H). The findings prove that APCs can carry reporter or therapeutic genes to the site of interest (here at the site of active angiogenesis) and magnetically labeled APCs will act as probe for cellular MRI. MRI and SPECT of control unlabeled non-transduced AC133+ progenitor cells (I, J)

**Figure 3:**
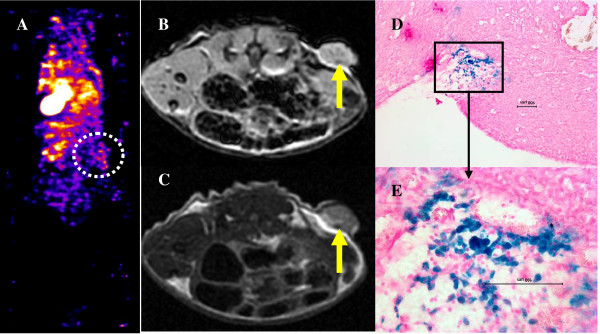
**Accumulation of magnetically labeled, non-transgenic APCs around the implanted tumor 7 days after IV administration**. (A) Coronal sections of SPECT data indicating non-significant activity of Tc-99m at the tumor site (within the white circular ROI). MRI shows low signal intensity areas at the periphery of the tumor (B, C, arrows), which are at the corresponding sites of iron positive cells detected by Prussian blue staining (D, E).

MR images clearly showed the presence of low signal intensity areas around and inside the tumors in mice that received iron labeled cells compared with the tumors that received PBS only or unlabeled AC133+ cells, indicating the accumulation of administered cells (Figures 2 and 3). The presence of iron labeled cells was also confirmed by PB staining (Figures 2 and 3).

Immunohistochemistry confirmed the accumulation of NIS positive cells at the corresponding sites, as detected by FITC labeled secondary antibodies (Figure 2G). These findings prove that APCs can carry reporter or therapeutic genes to the site of interest. In this case, they were found at the periphery of the tumors where active angiogenesis was observed. The accumulated cells also showed the expression of endothelial markers such as CD31 (Figure 2H).

### SPECT Analysis

**Figure 4:**
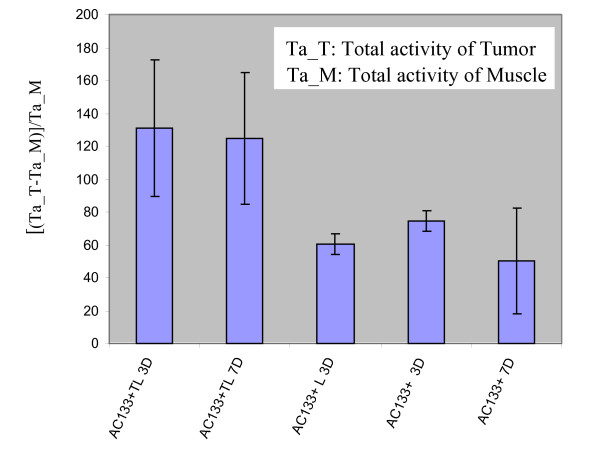
**Quantitative analysis of the ratio of total radioactivity of tumor and contra-lateral muscle**. There was no significant difference between total radioactivity of mice tumors injected by AC133 labeled transduced cells in 3 or 7 days (p-value > 0.05). There was a significant difference between total radioactivity of the tumor with injected ed labeled cells and non-transduced cells for both AC133+ only and AC133+ labeled cells (p-value < 0.05).

SPECT image analysis showed that the ratio of the total radioactivity (tumor/contralateral tissues) was significantly higher in animals that received transduced cells (Figures 2B, 2D) than those that received non-transduced cells (Figure 3A). A comparison of the ratio of radioactivity among the different groups of animals is shown in Figure 4. Significantly increased radioactivity ratios were observed in tumors of the animals that received magnetically labeled transgenic AC133+ cells compared to that of other groups both on days 3 and 7 (p < 0.05). There was no significant difference in radioactivity observed between days 3 and 7 in animals that received transgenic cells (p-value > 0.05). There were also no significant differences in the accumulated radioactivity in tumors observed among animals that received either labeled or unlabeled non-transgenic AC133+ cells. These findings indicate that the increased accumulation of radioactivity in tumors of animals that received transgenic cells is related to the expression of hNIS in the migrated and incorporated AC133+ cells. The hNIS bearing cells were also confirmed by immunohistochemistry.

Figure 4 reflects our observation of non-specific increased activity of Tc-99m at the site of tumors compared to the contralateral muscle in animals that received either only PBS or non-transduced unlabeled or labeled cells. This non-specific activity might be due to increased blood volume in the tumors and extravasations of Tc-99m. However, this non-specific activity was significantly lower than that found in tumors in animals that received transduced cells.

## Discussion

Experimental results confirmed our hypotheses that magnetically labeled transgenic APCs can be used as cellular probes for MRI and gene carrier systems for breast cancer. Gene delivery vehicle is a key tool in cancer gene therapy. Carrier vehicles are needed for the delivery of therapeutic genes to the target cancer cells. These vehicles should be specifically targeted to the cancer cells. Lack of an appropriate carrier in addition to the infiltrative nature of breast cancer is one of the main limitations of successful breast cancer gene therapy [29]. To control the release of administered viruses into circulation, genetically transformed cells are being considered for gene therapy for different tumors [30-35]. Due to its unique property to migrate to the pathological lesions, stem cells are unique vehicles for delivering therapeutic genes to the tumors, especially for glioma [36-39]. Rat neural stem cells expressing the cytosine deaminase gene, injected at a site distant from the primary tumor exhibit extensive migration and stable expression of the gene, indicating persistent ability to destroy tumor cells locally as well as distal from the main tumor mass or metastatic foci [40]. Mesenchymal stem cells, pluripotent bone marrow stromal cells, are also used to carry genes to glioma and are considered effective delivery vehicles [39,41-43]. APCs, which are a subpopulation of pluripotent hematopoietic stem cells showed active migration and incorporation into the neovasculatures of tumor when administered locally or systemically [17,27,44]. Ferrari et al. [45] have shown the migration and incorporation of HSV-tk transduced mouse APCs in subcutaneous tumor in a mouse model, however, they did not show incorporation of the transduced cells by *in vivo *imaging. Our results support the findings of previous reports that transgenic stem cells can migrate and accumulate at the sites of implanted tumors [46,47]. However, the most important aspect of our study was that the transgenic cells were tracked by *in vivo *imaging (both by MRI and SPECT). To the best of our knowledge, this is the first study to use different imaging modalities to show the migration and accumulation of administered transgenic stem cells. The findings of this proof-of-principle study pave the road for further studies in other tumor models.

In recent years, imaging techniques that enable efficient and non-invasive *in vivo *monitoring and tracking of transplanted cells have become central to the development of cell transplantation based therapeutic approaches. Recent studies using various disease models demonstrated that MRI can be used as a high resolution imaging technique for *in vivo *cell tracking [17-20]. Furthermore, MRI has been successfully used to track the migration and incorporation of intravenously injected, magnetically labeled APCs into the blood vessels in rodent tumor model [17,27]. MRI along with the adequate labeling reagent allow for monitoring of the entire time course of APC migration. They also can show possible accumulation of labeled cells in different tissues. *In vivo *detection and measuring of gene expression using reporter genes is another imaging ability. Reporter gene methods provide a unique opportunity to study biology in living subjects. They also allow for monitoring of physiological events in an intact environment. NIS is a membrane glycoprotein that mediates active iodide uptake in thyroid gland and some other tissues [48]. Human NIS (hNIS) gene expression has been shown with radioisotopes of iodine and Tc-99m-pertechnetate [49]. The hNIS has advantages over other reporter genes such as HSV1-tk and luciferase in that the hNIS gene product is innate and nonimmunogenic. It has limited endogenic tissue-specific expression and can mediate the uptake of chemically simple and available radiopharmaceuticals. We have shown that transduced APCs containing hNIS can easily be tracked and detected using Tc-99m and SPECT. This confirms that our cells are transduced by adenoviral vectors and hNIS is expressed in the APCs at the tumor sites. Tumor histology in addition to MRI analysis confirmed the presence of magnetically labeled and genetically transduced cells at the periphery of tumor where active angiogenesis was observed.

Limitations of this study are as follows. Viability of cells after viral transduction was one limitation, although our results were comparable to other published reports that used viral vectors for transduction of stem cells [50]. The overall transduction efficiency (we showed that 35% of our cells were transduced), even with higher ratio of viral particles, was low in our case using adenoviral vectors because of a limited presence of CAR receptors on hematopoetic cells [51]. However, the transduction was relatively stable for at least 12 days. The use of lentiviral or retroviral vectors would likely have produced more transduced APCs [52]. Future projects will utilize different effective viral vectors for relative stable transduction. We are currently optimizing the virus-to-cell ratio to get more viable cells after transduction and to get higher transduction efficiency. The timing of cell injection is another variable that should be optimized. Multiple injections at different time points in the same animal should also be considered to increase the delivery of genes to the tumor site.

### Future Potential

The stem cell gene delivery system has the potential to open a new window to the clinical cancer gene therapy. APCs can be collected from patient's own peripheral blood and bone marrow to avoid the immune system reaction. An important application of our gene delivery system is the delivery of suicidal genes to the cancer cells. In this approach, genes such as HSV-TK/acyclovir (ACV), ganciclovir (GCV), or herpes simplex virus-1-thymidine kinase (HSV-TK) convert a non-toxic pro-drug into a toxic drug. The bystander effect, a main phenomenon in this treatment modality, occurs when neighboring cancer cells, not specifically targeted with the suicidal gene, are also destroyed. The cell-to-cell transfer of phosphorylated GCV via gap junctions between HSV-TK transduced cells and neighboring non-infected cells is the primary mechanism for the bystander effect [53-55]. The efficacy of this protocol will be directly related to the gene/pro-drug combination and the target (cancer cells).

## Conclusion

MRI and SPECT images showed accumulation of administered APCs in the implanted breast cancer and expression of hNIS gene, respectively. Our study indicates that APCs can carry therapeutic genes even after systemic administration. Genetically transformed, magnetically labeled APCs can be used both as delivery vehicles and cellular probes for detecting *in vivo *migration and homing of cells by MRI. This method can be used in the future development of gene therapy approaches where genetically modified cells can be tracked in real time and *in vivo *by MRI in different disease processes.

## Methods

### AC133+ progenitor cells (APCs)

Fresh whole blood was obtained from the cord blood under IRB approved protocol with proper informed consent. Blood was collected in heparinized tubes. The blood was diluted 1:2 in phosphate buffered saline (PBS) plus 2 mM EDTA (Ethylenediaminetetraacetic Acid), layered onto lymphocyte separation medium (Ficoll, density 1.077 g/ml, ICN Biomedicals, Aurora, OH) and centrifuged for 30 minutes at 1,900 RPM, and at a temperature of 20°C (35 ml blood was very gently added onto 15 ml lymphocyte separation medium in every 50 ml sterile tube). Then, the white ring fraction (mononuclear cell layer) was transferred to a new 50 ml tube using a sterile Pasteur pipette. PBS was added twice and the tubes were centrifuged for 15 minutes at 1500 RPM at room temperature. After discarding the supernatant, the pellet was resuspended in 4 ml ACK lysine buffer to get rid of (for lysing) remaining erythrocytes. Mononuclear cells (MNCs) were incubated in the ACK lysine buffer for no more than 3 minutes on ice. After 3 minutes, 20 ml PBS was added to the solution, the cells were washed twice (centrifuge at 1200 RPM) with PBS. MNCs were subjected to immunomagnetic separation using AC133 Cell Isolation Kit (Miltenyi Biotech; Auburn, CA; ), according to manufacturer's instruction [56,57]. Briefly, MNCs were incubated for 45 minutes on ice with an FcR-blocking reagent (human IgG) and AC133 MicroBeads. These microbeads are conjugated with monoclonal mouse anti-human antibodies against AC133. After washing with PBS plus 2 mM EDTA, the labeled cells were filtered through a 30-μm nylon mesh and loaded onto a column that was installed in a magnetic field. Trapped cells were eluted after the column was removed from the magnet. The collected cells were named AC133+ progenitor cells (APCs). APCs were grown in stemline II media (Sigma, St. Louise, MO) supplemented with 10 ng/ml of thrombopoietin (TPO), 40 ng/ml of FLT3 and 40 ng/ml of stem cell factor (SCF). Initially, the cells were suspended in media at 1 × 10^6 ^per ml and grown in 5% CO_2_/95% air at 37°C in a humidified incubator with fresh media added on every third day. The cells were propagated for 7–10 days. On the day of labeling, the cells were harvested and resuspended at a concentration of 4 × 10^6 ^cells per ml. Collected cells were analyzed at different days in culture for their phenotypical expression. There were gradual declines in the expression of CD133 and CD34, but the majority of cells were positive for CD31, CXCR4 and CD117. Markers of lymphocytic (CD3, CD19) and monocytic (CD14) lineages were absent.

### Breast Cancer Cells (MDA-MB-231)

Breast cancer cells (MDA-MB-231, ATCC, VA) were cultured in 75-cm^2 ^tissue culture flasks with Dulbecco's modified Eagle's medium (DMEM) supplemented with 10% fetal bovine serum, penicillin (100 IU/mL), and streptomycin (100 μg/mL) until they were 80–90% confluent.

### Tumor Implantation

All animal studies were approved by the institutional animal care and user committee at Henry Ford Health System. Female nude mice (total 48 mice) were used for implantation of tumor cells. Tumor cells (human breast cancer), cultured in-vitro were harvested by trypsinization and centrifugation, washed twice with PBS buffer, and then resuspended in cell culture serum free media at a concentration of 6 × 10^7 ^cells/ml. The animals received one subcutaneous injection of 50 μl of cell suspension (3 million cells) in the right hind flank. These tumor cells are efficient in making xenografted tumors with almost 90% efficiency and show marked vascularity with less central necrosis. Tumors were measured twice a week after implantation in two dimensions. All animals received intravenous injection of APCs when the tumor measured between 0.5 to 1 cm diameter in the largest dimension.

### Transduction of APCs with Adenoviral Vector Containing HSV-tk and hNIS Genes

**Figure 5:**
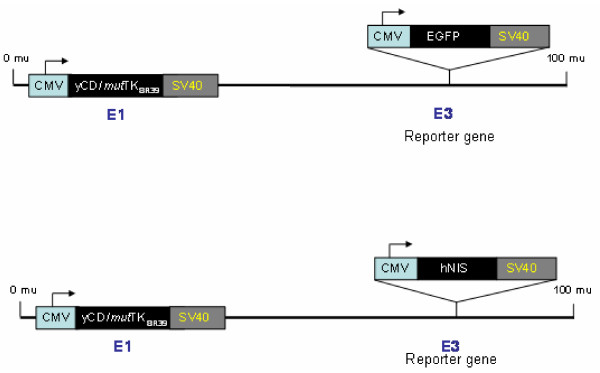
**Adenovirus design to detect transfected efficiency by flowcytometric (EGFP) and nuclear medicine (hNIS using Tc-99m) techniques**. This figure is printed with the permission of Kenneth N Barton from the Radiation Oncology Department of Henry Ford Hospital.

An adenoviral vector carrying either the hNIS or EGFP gene was used to transduce cells (Figure 5). Both vectors also contained the HSV-tk gene, which can be used as a suicidal gene for therapeutic purposes. hNIS and EGFP acted as reporter genes and their activities were determined by Tc-99m pertechnetate (Tc-99m) SPECT and flowcytometer, respectively. Cell to viral particle ratio was maintained at 1:1000. Cells were at a concentration of 10 million per ml for initial 1 hour and then diluted ×10 with complete culture media. Transduction efficiency was determined on day 3 by flowcytometric analysis (using EGFP as reporter gene) and fluorescent microscope. These transgenic cells were magnetically (FePro) labeled on day 4 after transduction.

### Labeling of Cells with Ferumoxide-Protamine Sulfate (FePro) Complex

The commercially available, FDA-approved SPIO, ferumoxide suspension, (Feridex IV ^®^, Berlex Laboratories, Inc, Wayne, New Jersey) contains particles approximately 80–150 nm in size and has a total iron content of 11.2 mg/ml. Protamine sulfate (American Pharmaceuticals Partner Inc. Schaumburg, IL), supplied at 10 mg/ml, was prepared as a fresh stock solution of 1 mg/ml in distilled water at the time of use. Ferumoxide, at a concentration of 100 μg/ml, was put into a mixing flask or tube containing serum-free RPMI 1640 medium (Biosource, Camarillo, CA) containing non-essential amino acid, sodium pyruvate, and L-glutamine. Protamine sulfate was then added to the solution at a concentration of 3–4.5 μg/ml of mixing media. The solution containing ferumoxide and protamine sulfate was mixed for 30 seconds with intermittent hand shakings. After 30 seconds, the FePro complex was added directly to the cells, incubated for 2–3 hours, and then an equal volume of the complete medium was added to the cells, for a final concentration of 50 μg ferumoxide/ml of medium. The cell suspension was then incubated overnight. After overnight incubation, the cells were washed three times and then resuspended in PBS.

### Measuring Intracellular Iron

Intracellular iron concentration was measured based on our previously published method by UV/VIS spectrophotometry [58]. In brief, cell suspensions with known cell density were demineralized in 1 ml of 5 Mol/L hydrochloric acid for 4 hours. Then, the color of the mixture was read with a UV/VIS spectrometer at 351 nm wave-length. The absorption value was normalized to a standard curve generated from a known solution of ferumoxide. To determine the very low concentration of iron in the sample, the iron concentration was measured using this method and is expressed as an average picogram of iron/cell. Currently, this method is used in our laboratory to determine the very low concentration of iron in the sample. The commercially available kit for measuring iron concentration was not sensitive enough to detect such low iron concentrations (< 1 μg/ml).

### Determination of Labeling Efficiency

After incubation with FePro, the cells were washed 3 times in the presence of heparin sulfate to remove excess FePro and transferred to cytospin slides. Cells were fixed and stained with Perl's reagent for Prussian blue (PB) staining. FePro labeling efficiency was determined by manually counting PB stained and unstained cells using a microscope at 40× magnification. Cells were considered PB positive if intracytoplasmic blue granules were detected. The percentage of labeled cells was determined from the average of 5–10 high-powered fields.

### Cellular viability of FE-Pro labeled cells

After labeling, the cells were washed at least twice with sterile PBS and then resuspended in PBS at a concentration of 3 × 10^7 ^cells/ml. A small aliquot of cells were mixed with trypan blue dye and checked under a microscope to determine cell viability.

### Intravenous Administration of hNIS Transduced and FePro Labeled APCs

Either hNIS transduced and FePro labeled APCs or control APCs (without transduction and labeling) were administered intravenously into tail vein of mouse when the tumor grew to 0.5 cm in size (along the largest dimension). A total of three million APCs in 200 μl was administered intravenously. Following administration of APCs, mice underwent MRI and Tc-99m SPECT scanning to determine the migration and accumulation of FePro labeled and transgenic (containing hNIS) cells, respectively, on days 3 and 7 after cell administration.

### Magnetic Resonance Imaging Study

#### Preparation

Anesthesia was induced and maintained during all MRI studies using an inhalation mixture of isoflurane (3% for induction and 0.5–1.5% for maintenance) in a 2:1 N_2_O/O_2 _gas mixture, with the animal breathing spontaneously. After induction, the animal was placed in a custom made MRI compatible Plexiglas holder that was equipped a nose cone for the delivery of gasses and a heating pad. After securing the animal's nose inside the mask, the hindquarters of the animal were positioned and secured to the holder, a rectal probe was inserted for temperature monitoring, and the holder was placed inside the imaging coil. Next, the animal holder/imaging coil assembly was placed inside the magnet, isoflurane was reduced to 0.5–1.5%, and the animal's core temperature maintained at 37°C using a feedback controlled water bath.

#### MRI Systems

MRI experiments were conducted using either a 7 Tesla (7 T) or a 3 Tesla (3 T) MRI system. The 7 T system is a 12 cm (clear bore) magnet (Magnex Scientific, Inc., Abingdon, UK) interfaced to a Bruker console (Bruker Biospin MRI, Inc, Billerica, MA) with actively shielded gradients of 25 gauss/cm and 100 μs rise time. A 5 cm diameter quadrature birdcage RF coil tuned to 300 MHz is used for RF transmission and reception. The 3 T system is a whole body magnet (Signa Excite, GE health, Milwaukee, WI) that uses dedicated small animal coils (Doty scientific) for animal imaging. Once inside the magnet, the position of the animal was adjusted so that the central imaging slice coincided with the center of the tumor.

#### T2 Measurement using 7 T System

Multi-slice T2-weighted images were acquired using a multi-echo spin-echo sequence with a fixed repetition time (TR) and progressively incremented echo times (TE) [TR/TE = 3000/12, 24, 36, 48 ms]. Imaging parameters were as follows: FOV = 32 mm, matrix = 256 × 256, 17 slices (interleaved slice acquisition), slice thickness = 1 mm. The total scan time was approximately 13 minutes.

#### Susceptibility Weighted Imaging (SWI) using 7 T System

This is a specialized 3D gradient-echo sequence, very sensitive to the presence of iron and can detect iron labeled cells. The method uses TR/TE = 50 ms/6.7 ms, flip angle = 15 degrees, BW = 50 KHz, 32 × 32 × 16 mm^3 ^FOV, 256 × 256 × 64 matrix (resolution of 125 × 125 × 250 mm^3^), 2 averages with flow compensation applied in all three directions. The total scan time for this 3D data set was approximately 27 minutes.

#### T2 Measurement using 3 T System

Multi-slice T2 images were acquired using a Carr, Purcell, Meiboom, and Gill (CPMG) multi-echo spin-echo sequence with a fixed repetition time (TR) and progressively incremented echo times (TE) [TR/TE = 2400/15, 30, 45, 60 ms]. The imaging parameters were as follows: FOV = 35 mm, matrix = 160 × 128, averages = 3, 15 slices, slice thickness = 1 mm. The total scan time was approximately 5 minutes.

#### T2* Measurements using 3 T System

Multi-slice T2* images were acquired using a multi-echo gradient-echo sequence with a fixed repetition time (TR) and progressively incremented echo times (TE) [TR/TE = 600/10, 15, 20, 30 ms]. The imaging parameters were as follows: FOV = 35 mm, matrix = 160 × 128, averages = 4, 15 slices, slice thickness = 1 mm. The total scan time was approximately 8 minutes.

#### FIESTA using 3 T System

This is a specialized 3D gradient-echo sequence, very sensitive to the presence of iron and can detect iron labeled cells. The method uses TR/TE = 11–15 ms/3–7 ms, flip angle = 40 degrees, BW = 10 kHz, 60 × 60 × 40 mm^3 ^FOV, 300 × 300 × 200 matrix (resolution of 200 × 200 × 200 mm^3^), 2 averages. The total scan time for this 3D data set was approximately 15 minutes.

### SPECT Study

Within 24 hours after MRI, animals underwent SPECT studies using Tc-99m to determine the status of genetically transformed cells at the sites of tumors. Animals were anesthetized using ketamine/xylazine (100/15 mg/kg) and received 1 mCi of Tc-99m through tail vein injection in a volume of 200 μl. 60 minutes after Tc-99m injection, animals were wrapped with padded sheet to keep them warm and placed in the imaging holder under anesthesia. SPECT images were acquired with a dedicated PRISM 3000 gamma camera fitted with mutli-pinhole mouse collimator, 360 degree rotation with 36 degree increments, 180 sec per projection, using 256 × 256 matrices with a field of view of 4 × 6 cm. The total time required for acquiring SPECT images was about 31 minutes.

#### Euthanasia and Histological Analysis

Immediately after SPECT imaging, the animals were anesthetized using pentobarbital (100 mg/kg intravenous or intraperitoneal) and sacrificed. The tissues were fixed for histological analysis by perfusion with saline, for vascular washout, followed by paraformaldehyde. The radioactive fluids were collected and contained in a shielded area to decay. The animals were euthanized either with 100 mg/kg of pentobarbital administration (intravenous or intraperitoneal). The whole tumor and part of surrounding tissues (muscles and skin) were collected for histochemical determination of iron labeled cells using PB staining and markers of endothelial cells, such as CD31 and vWF using human specific antibodies (BD Pharmingen and DakoCytomation respectively). Expression of hNIS in accumulated cells was determined by staining with anti-hNIS antibody (Genetex, TX, USA).

#### Study Design

There were four groups of animals (n = 6/group) for each time point (days 3 and 7 after IV administration of cells): 1) controls injected with PBS: 2) tumors injected with unlabeled APCs; 3) tumors injected with magnetically labeled APCs; and 4) tumors injected with transduced and magnetically labeled APCs. The total number of animals used in the study was 48.

#### Image and Statistical Analysis

Eigentool image analysis software (Henry Ford Hospital, Detroit, MI, USA) was used to analyze MRI images. Eigentool has a comprehensive set of functions for displaying, restoring, enhancing, and analyzing images [59-61]. Statistical analysis of SPECT images was performed with SPSS software (version 13; SPSS Institute Inc.) for Microsoft Windows. The data were analyzed with two-way analysis of variance and general linear models. The significance level was set at 0.05 and statistical analysis of the tumor-to-muscle ratio was performed with constructed SPECT images. ImageJ software was also used for SPECT analysis. Tumor volume was defined on the SPECT images by drawing ROIs around the tumor on each slice and the total activity was recorded. The same volume was used to compute the total activity from the contra-lateral muscle region.

The percentage of change in the total activity (ΔTa%) was calculated using:

(1) ΔTa% = 100 × (TaT-TaM)/TaM

where TaT is the total activity of the tumor and TaM is the total activity of the contralateral muscle for the same volume as that of the tumor.

## Abbreviations

APC: AC133+ progenitor cells; SPIO: superparamagnetic iron oxide; MRI: Magnetic resonance imaging; hNIS: human sodium iodide symporter; FePro: ferumoxides-protamine sulfate; SPECT: single photon emission computed tomography; FDA: Food and Drug Administration; PBS: phosphate buffered saline; EDTA: Ethylenediaminetetraacetic Acid; TPO: thrombopoietin, SCF: stem cell factor; MNCs: Mononuclear cells; DMEM: Dulbecco's modified Eagle's medium; PB: Prussian blue; ACV: acyclovir; GCV: ganciclovir; HSV-TK: herpes simplex virus-1-thymidine kinase; EGFP: Enhanced Green Fluorescent Proteins.

## Authors' contributions

AMR carried out the experimental design, isolation of stem cells, tumor implantation, cell transduction and labeling, MRI and SPECT imaging, data analysis, and initial manuscript preparation. ASMI participated in the isolation of stem cells, tumor implantation, stem cell injection, and histology. BJ participated in the cell transduction and labeling. RAK participated in the MRI and manuscript preparation. ASA provided intellectual support and participated in the experimental design, analysis of MRI and SPECT, and manuscript preparation. HSZ provided intellectual support and helped with image processing and manuscript preparation. All authors read and approved the final manuscript.
